# Diastolic wall strain: a simple marker of abnormal cardiac mechanics

**DOI:** 10.1186/1476-7120-12-40

**Published:** 2014-10-03

**Authors:** Senthil Selvaraj, Frank G Aguilar, Eva E Martinez, Lauren Beussink, Kwang-Youn A Kim, Jie Peng, Daniel C Lee, Ateet Patel, Jin Sha, Marguerite R Irvin, Donna K Arnett, Sanjiv J Shah

**Affiliations:** Division of Cardiology, Department of Medicine, Northwestern University Feinberg School of Medicine, 676 N. St. Clair St., Suite 600, Chicago, IL 60611 USA; Department of Preventive Medicine/Biostatics, Northwestern University Feinberg School of Medicine, Chicago, IL USA; Departments of Epidemiology and Biostatistics, School of Public Health, University of Alabama Birmingham, Birmingham, AL USA

**Keywords:** Strain, Speckle-tracking, Echocardiography, Cardiac mechanics, Diastolic dysfunction, Systolic dysfunction

## Abstract

**Background:**

Diastolic wall strain (DWS), defined using posterior wall thickness (PWT) measurements from standard echocardiographic images (DWS = [PWT(systole)-PWT(diastole)]/PWT(systole)), has been proposed as a marker of left ventricular (LV) diastolic stiffness. However, the equation for DWS is closely related to systolic radial strain, and whether DWS is associated with abnormal cardiac mechanics (reduced systolic strains and diastolic tissue velocities) is unknown. We sought to determine the relationship between DWS and systolic and diastolic cardiac mechanics.

**Methods:**

We calculated DWS and performed speckle-tracking analysis in a large population- and family-based study (Hypertension Genetic Epidemiology Network [HyperGEN]; N = 1907 after excluding patients with ejection fraction [EF] < 50% or posterior wall motion abnormalities). We measured global longitudinal, circumferential, and radial strain (GLS, GCS, and GRS, respectively) and early diastolic (e’) tissue velocities, and we determined the independent association of DWS with cardiac mechanics using linear mixed effects models to account for relatedness among study participants. We also prospectively performed receiver-operating characteristic (ROC) analysis of DWS for the detection of abnormal cardiac mechanics in a separate, prospective validation study (N = 35).

**Results:**

In HyperGEN (age 51 ± 14 years, 59% female, 45% African-American, 57% hypertensive), mean DWS was 0.38 ± 0.05. DWS decreased with increasing comorbidity burden (β-coefficient -0.013 [95% CI -0.015, -0.011]; P < 0.0001). DWS was independently associated with GLS, GCS, GRS, and e’ velocity (adjusted P < 0.05) but not LV chamber compliance (EDV_20_, P = 0.97). On prospective speckle-tracking analysis, DWS correlated well with GLS, GCS, and GRS (R = 0.61, 0.57, and 0.73, respectively; P < 0.001 for all comparisons). C-statistics for DWS as a diagnostic test for abnormal GLS, GCS, and GRS were: 0.78, 0.79, and 0.84, respectively.

**Conclusions:**

DWS, a simple parameter than can be calculated from routine 2D echocardiography, is closely associated with systolic strain parameters and early diastolic (e’) tissue velocities but not LV chamber compliance.

## Background

Left ventricular (LV) diastolic dysfunction is common in the general population, and is associated with incident heart failure and increased mortality [1, 2]. The pathophysiology of diastolic dysfunction is complex, but can be simply described as impaired LV myocardial relaxation and/or increased LV stiffness, both of which can lead to increased LV filling pressures at rest or with exercise. Although Doppler echocardiography is able to detect impaired LV relaxation and elevated LV filling pressures quite well, the detection of reduced LV compliance (i.e., increased LV stiffness) has proven to be more difficult, requiring invasive pressure-volume analysis for calculation of the end-diastolic pressure-volume relationship (EDPVR).

Recently, a non-invasive, load-independent, and reproducible estimator of LV stiffness using 2-dimensional (2D) echocardiography, namely diastolic wall strain (DWS), has been proposed [3, 4]. DWS, an extension of linear elastic theory, uses the difference between posterior wall thickness in systole (PWTs) and diastole (PWTd) to approximate LV stiffness [4]. According to the theory, decreased wall thinning during diastole reflects reduced LV compliance and distensibility, and thus, increased LV stiffness.

However, DWS, as it name implies, is closely related to systolic strain. DWS, calculated as [(PWTs) – (PWTd)]/(PWTs), can be simplified purely in terms of myocardial (wall) strain, defined as [(PWTs) – (PWTd)]/(PWTd). By rearranging the two equations, DWS can be expressed as [(wall strain)/(1 + wall strain)] [4]. Takeda et al., whose work validated the use of DWS, failed to demonstrate a correlation between tissue-Doppler derived strain and DWS [4]. However, speckle-tracking echocardiography holds several advantages over tissue-Doppler in measuring strain, including superior reliability, less angle dependence, and greater ability to differentiate normal from dysfunctional myocardial segments [5].

Though Takeda et al. demonstrated that DWS correlates moderately well (R = -0.47, P < 0.05) with invasive measurements of myocardial stiffness [4], uncertainty still remains in how best to interpret this new marker. A recent editorial has framed the debate [6]. DWS can conversely be thought of as an index of wall thickening, not just wall thinning, and may therefore measure LV systolic function. Further, DWS should theoretically correlate well with radial strain, which is itself a systolic index. That DWS may actually correlate well with both systolic and diastolic indices suggests, in fact, that DWS is rather an overall marker of myocardial health and performance.

The evaluation of echocardiograms from the Hypertension Genetic Epidemiology Network (HyperGEN) study permits a robust assessment of the relationship between DWS and cardiac mechanics. HyperGEN, conducted from 1996–2002, originally sought to determine the genetic basis for familial hypertension. Advantages of the HyperGEN study include a bi-racial sample of approximately 3600 participants, comprehensive clinical and laboratory data collection, and 2D/Doppler echocardiographic data [7]. Though echocardiograms were performed at a time prior to digital storage, we have successfully implemented a technique to convert analog echocardiograms to digital format, permitting post-hoc speckle-tracking with the subsequent determination of cardiac mechanics [8].

Therefore, we sought to determine the association of DWS with LV systolic and diastolic mechanics. We hypothesized that DWS correlates with both systolic and diastolic measures of LV performance. We further hypothesized that reduced DWS is associated with systolic LV mechanics (i.e., decreased LV strain), even when adjusting for LV geometry and echocardiographic indices of filling pressures and myocardial relaxation.

## Methods

### Study population

HyperGEN, part of the National Institutes of Health Family Blood Pressure Program (FBPP), is a cross sectional-study consisting of five U.S. sites, while four participated in an ancillary echocardiographic study (Salt Lake City, Utah; Forsyth County, NC; Minneapolis, Minnesota; and Birmingham, Alabama). The goal of HyperGEN was to identify and characterize the genetic basis of familial hypertension; complete details of the HyperGEN study design have been reported previously [7]. Study eligibility required a diagnosis of hypertension prior to the age of 60 years and at least one sibling willing to participate in the study. Hypertension was defined by an average systolic blood pressure ≥ 140 mmHg or an average diastolic blood pressure ≥ 90 mmHg (on at least 2 separate clinic visits) or by self-reporting treatment for hypertension. Age-matched normotensive patients were also enrolled as control subjects. Individuals with a history of type 1 diabetes mellitus or severe chronic kidney disease were excluded due to the high risk of secondary forms of hypertension. None of the study participants had symptomatic heart failure. All HyperGEN study participants gave written informed consent, and the HyperGEN study was approved by each study site’s local institutional review board. For the present study, we restricted our initial population to 2150 participants based upon videotapes available at the time of analysis, sampled at random from all four participating centers.

### Clinical characteristics

Demographic, clinical, and laboratory data were collected during the initial HyperGEN visit. Height, weight, blood pressure, and waist circumference were measured by a technician. Histories of myocardial infarction, transient ischemic attack, or stroke were obtained by self-report. Diabetes mellitus was defined by fasting glucose ≥ 126 mg/dl, use of hypoglycemic medication, or a self-reported history. Obesity was defined by a body mass index ≥ 30 kg/m^2^. Chronic kidney disease was defined by an estimated glomerular filtration rate ≤ 60 ml/min/1.73 m^2^.

### Echocardiography

Doppler, 2D, and M-mode echocardiograms were acquired using standardized acquisition protocols and stored in analog format (high grade, medical quality videocassette tapes) at the time of visit [9, 10]. Cardiac structure and function were quantified as recommended by the American Society of Echocardiography [11, 12]. LV mass was calculated and indexed to body surface area, and LV hypertrophy was defined by an LV mass index > 95 g/m^2^ in women or > 115 g/m^2^ in men. DWS was calculated as [(PWTs) – (PWTd)/(PWTs)] [3, 4]. Participants with missing posterior wall thickness values (N = 81), EF < 50% (N = 118), and posterior wall motion abnormalities (N = 44) were excluded from analyses, consistent with criteria from previous studies [3, 4].

### Digitization and interpretation of image quality

Archived echocardiograms in analog format were converted to digital format using the TIMS 2000 DICOM System (Foresight Imaging, Chelmsford, MA). Cine loops of 2–4 cardiac cycles from the parasternal short axis (papillary muscle level) and apical four chamber views were digitized at a frame rate of 30 to 40 frames per second and stored offline in DICOM format. Each study was assessed for image quality by an experienced operator, blinded to all other clinical and echocardiographic data, using a 4-point scale based on the degree of endocardial border visualized (1 = 0-25%; 2 = 25%-50%; 3 = 50%-75%; 4 = 75%-100%), similar to scales used previously [13, 14].

### Two-dimensional speckle-tracking analysis

Digitized cine loops were analyzed using 2D wall motion tracking software [2D Cardiac Performance Analysis (CPA), TomTec v4.5, Unterschleisshein, Germany]. After isolating the highest quality cardiac cycle, the endocardial and epicardial borders were traced at end-systole in each view. Computerized speckle-tracking analysis was performed, and endocardial and epicardial border tracings were manually adjusted to optimize tracking.

Components of LV strain (global longitudinal, radial, and circumferential) and tissue velocities (septal and lateral e’) values were recorded. For ease of display, all strain values were converted to absolute values (i.e., longitudinal and circumferential strain values were converted from negative to positive values). Lower absolute strain values, lower e’ tissue velocities, and higher E/e’ ratio were used to indicate worse cardiac function. Speckle-tracking tissue velocities were used instead of tissue Doppler velocities since HyperGEN echocardiograms were acquired at a time prior widespread use of tissue Doppler technology. However, we converted speckle-tracking echocardiography-derived tissue velocities into tissue Doppler velocities using a regression equation constructed from a separate cohort of 100 prospectively studied patients referred to the Bluhm Cardiovascular Institute (Northwestern Memorial Hospital, Chicago, IL) for clinically indicated echocardiography with tissue Doppler imaging (Philips ie33 [Philips Medical Systems, Andover, MA] or GE Vivid 7 [GE Medical Systems, Milwaukee, WI]). All patients gave written, informed consent, and the Northwestern University Institutional Review Board approved the study of these patients. These echocardiograms were analyzed using the same software used in the analysis of HyperGEN participants (2D Cardiac Performance Analysis [TomTec, Unterschleisshein, Germany]), and velocities from the 2 methods (speckle-tracking vs. tissue Doppler) were compared. A regression equation was constructed for each tissue velocity [(septal tissue Doppler e’ velocity = 1.39*(speckle-tracking tissue e’ velocity) + 1.89 cm/s; lateral tissue Doppler e’ velocity = 1.47*(speckle-tracking tissue e’ velocity) + 5.67 cm/s)]. A validation of the digitization and speckle-tracking techniques employed here has been published elsewhere [8].

### Calculation of the single-beat end-diastolic pressure volume relationship

In order to compare DWS to a measure of LV chamber compliance (LV EDPVR), we calculated LV end-diastolic stiffness as the slope of the EDPVR on pressure-volume analysis. The single-beat method was employed, and we calculated EDV_20_ (the predicted LV end-diastolic volume at an idealized LV end-diastolic pressure of 20 mmHg) based on mean α and β coefficients as previously described [15]. In order to use this method, an estimate of diastolic filling pressure (i.e. E/e’ ratio) is required. The lateral E/e’ ratio (which is preferred over the septal E/e’ ratio in participants with preserved ejection fraction) [16] was converted into LV filling pressure using a previously published formula [17].

### Validation study – prospective digital speckle-tracking echocardiography

To determine the clinical utility of DWS for the detection of abnormal strain parameters, we conducted an additional prospective validation study using digital speckle-tracking echocardiography-derived strain measurements. The validation cohort consisted of patients (N = 35) recruited from the Bluhm Cardiovascular Institute echocardiography laboratory. Each patient underwent echocardiography (GE Vivid 7) for research purposes using a pre-defined protocol, which included dedicated, zoomed-in views of the LV in the parasternal short axis and apical 4-, 3-, and 2-chamber views. The sector width and depth were minimized to ensure an adequate frame rate (50–70 fps). PWT measurements and speckle-tracking analysis were performed offline using EchoPAC software (GE Medical Systems, Milwaukee, WI). The speckle-tracking and DWS measurements were made > 3 months apart and both sets of measurements were made in a blinded fashion. All patients enrolled in the validation study provided written, informed consent, and the study was approved the Northwestern University Institutional Review Board.

### Statistical analysis

Clinical characteristics, laboratory data, and both conventional echocardiographic parameters and speckle-tracking parameters are displayed for the total HyperGEN cohort. Continuous data are presented as mean ± standard deviation. Categorical variables are presented as a count and percentage.

We evaluated intra- and inter-observer reliability in a randomly selected sample of 95 HyperGEN study participants. These echocardiograms were analyzed by 2 independent readers, blinded to their previous measurements, the other reader’s measurements, and all other data. Intra-observer measurements were performed one month after initial measurement. We evaluated the reproducibility of speckle-tracking measurements by calculating intra-class correlation coefficient, mean bias (using Bland-Altman analysis), and coefficient of variation. These data have been previously published [18].

In HyperGEN, we performed Pearson correlation analyses to determine the relationship between DWS and several systolic and diastolic echocardiographic indices. Next, we created multivariable models to determine the independent association between DWS and cardiac mechanics using mixed-effects linear regression, thereby accounting for relatedness among HyperGEN participants. Further models were constructed that additionally adjusted for indices of impaired relaxation (tissue e’ velocity, averaged from the septal and lateral values) and elevated filling pressures (E/e’ ratio, similarly averaged from the septal and lateral values) to determine whether DWS is independently associated with systolic cardiac mechanics beyond its association with these diastolic parameters. β-coefficients were reported per 0.01-unit change in DWS.

Covariates entered into the baseline multivariable model (besides family membership) included speckle-tracking analyst, image quality, and field center. Covariates selected for inclusion into additional regression models were based on a combination of clinical relevance (pre-specified based on face validity) and association with DWS in previous studies. These additional covariates (beyond the baseline model) included age, sex, LV mass index, ejection fraction, and wall motion score index. A two-sided p-value < 0.05 was considered statistically significant.

For the prospective validation study, we first created scatterplots and calculated Pearson correlation coefficients to determine the correlation between DWS and strain measurements. We then used published data on GLS, GCS, and GRS [19] to determine abnormal values for these parameters (GLS < 12.5%, GCS < 15%, or GRS < 35%.), and stratified the validation study patients into normal and abnormal groups by each strain parameter. Finally, we conducted receiver-operating characteristic (ROC) analyses to determine the area under the ROC curve (i.e., c-statistics) for DWS as a diagnostic test for the detection of abnormal systolic strain.

All statistical analyses were performed using Stata 12 software (StataCorp, College Station, TX).

## Results

### Characteristics of the HyperGEN study participants

Descriptive characteristics of the study sample from HyperGEN are displayed in Table 1. As stated above, from the original cohort of 2150 participants, individuals with missing DWS values (N = 81), EF < 50% (N = 118), and posterior wall motion abnormalities (N = 44) were excluded, leaving 1907 participants for analysis. Participants were sampled from all 4 participating sites, representing 1007 unique families. The mean age was 51 ± 14 years and 59% were female. The distribution of ethnicities was largely biracial and nearly even (54% white, 45% African American, and 1% other). Comorbidities were common, and medication use reflected standard therapies used in the comorbidities detailed in Table 1. Blood pressure was relatively well controlled (126 ± 20/72 ± 11 mmHg), and most study participants were obese (mean body mass index 31 ± 7 kg/m^2^).

**Table 1:** **Clinical characteristics of the HyperGEN study sample**

Characteristic	All HyperGEN participants (N = 1907)
Age, y	51 ± 14
Female, n (%)	1129 (59)
Ethnicity, n (%)	
● White	1032 (54)
● African-American	867 (45)
● Other	7 (1)
Recruiting center, n (%)	
● Birmingham, Alabama	531 (28)
● Minneapolis, Minnesota	376 (20)
● Forsyth County, North Carolina	537 (28)
● Salt Lake City, Utah	463 (24)
Comorbidities, n (%)	
● Hypertension	1085 (57)
● Obesity	897 (47)
● Diabetes mellitus	314 (16)
● Chronic kidney disease	158 (8)
● Myocardial infarction	83 (4)
● Transient ischemic attack or stroke	77 (4)
Medications, n (%)	
● Anti-hypertensive medication	950 (50)
● Angiotensin-converting enzyme inhibitor	381 (20)
● Angiotensin receptor blocker	51 (3)
● Alpha blocker	145 (8)
● Beta-blocker	245 (13)
● Calcium channel blocker	417 (22)
● Loop diuretic	108 (6)
● Thiazide diuretic	244 (13)
● Oral hypoglycemic	195 (10)
● Insulin	69 (4)
● Lipid lowering medication	163 (9)
● Statin	144 (8)
Physical examination:	
● Systolic blood pressure, mm Hg	126 ± 20
● Diastolic blood pressure, mm Hg	72 ± 11
● Body mass index, kg/m^2^	31 ± 7
● Waist circumference, cm	102 ± 16
Laboratory data:	
● Sodium, mEq/L	142 ± 2
● Creatinine, mg/dl	0.97 ± 0.28
● Estimated glomerular filtration rate, ml/min/1.73 m^2^	85 ± 20
● Fasting glucose, mg/dl	105 ± 43
● Total serum cholesterol, mg/dl	197 ± 39
● High density lipoprotein, mg/dl	51 ± 15
● Low density lipoprotein, mg/dl	119 ± 34

2D, Doppler, and speckle-tracking echocardiographic parameters are displayed in Table 2. Average values for LV structural parameters fell within normal limits, though roughly one-sixth (17%) had evidence of LV hypertrophy based on sex-specific cut-offs for elevated levels of LV mass index. Images upon which speckle-tracking was performed were generally of high quality. In the parasternal short-axis and apical four chamber views, 86% and 97% of images had an image quality score ≥ 2, respectively. Interobserver and intraobserver reliability data was excellent for all parameters (as shown previously [18]).

**Table 2:** **Echocardiographic characteristics the HyperGEN study sample**

Parameter	All patients (N = 1907)
**2D/Doppler echocardiographic parameter**	
LV end-systolic volume, ml*	48 ± 15
LV end-diastolic volume, ml*	127 ± 27
LV ejection fraction, %*	63 ± 6
LV midwall shortening, %*	17.9 ± 1.8
Mitral valve deceleration time, ms	204 ± 58
E/A ratio†, ‡	1.59 ± 0.46
Isovolumic relaxation time, ms	80 ± 18
Left atrial diameter, cm	3.4 ± 0.5
Interventricular septal wall thickness, cm	1.3 ± 0.1
LV mass index, g/m^2^ *	84 ± 19
LV hypertrophy, n (%)*	317 (17)
Posterior wall thickness at end-systole, cm	1.37 ± 0.14
Posterior wall thickness at end-diastole, cm	0.85 ± 0.12
Diastolic wall strain, %	0.38 ± 0.05
**Speckle-tracking echocardiographic parameter**	
Global radial strain, %	27.0 ± 11.7
Global circumferential strain, %	21.0 ± 5.1
Global longitudinal strain, %	14.8 ± 3.5
Septal e’ velocity, cm/s	7.1 ± 1.8
Lateral e’ velocity, cm/s	10.5 ± 2.1
Average e’ velocity, cm/s	8.8 ± 1.8
Septal E/e’ ratio†	10.6 ± 3.4
Lateral E/e’ ratio†	7.0 ± 1.9
Average E/e’ ratio†	8.7 ± 2.5

*LV = Left ventricular; †*E* = Early mitral inflow velocity; ‡*A* = Late (atrial) mitral inflow velocity.

### Correlation between DWS and echocardiographic systolic and diastolic indices in HyperGEN

**Figure 1:**
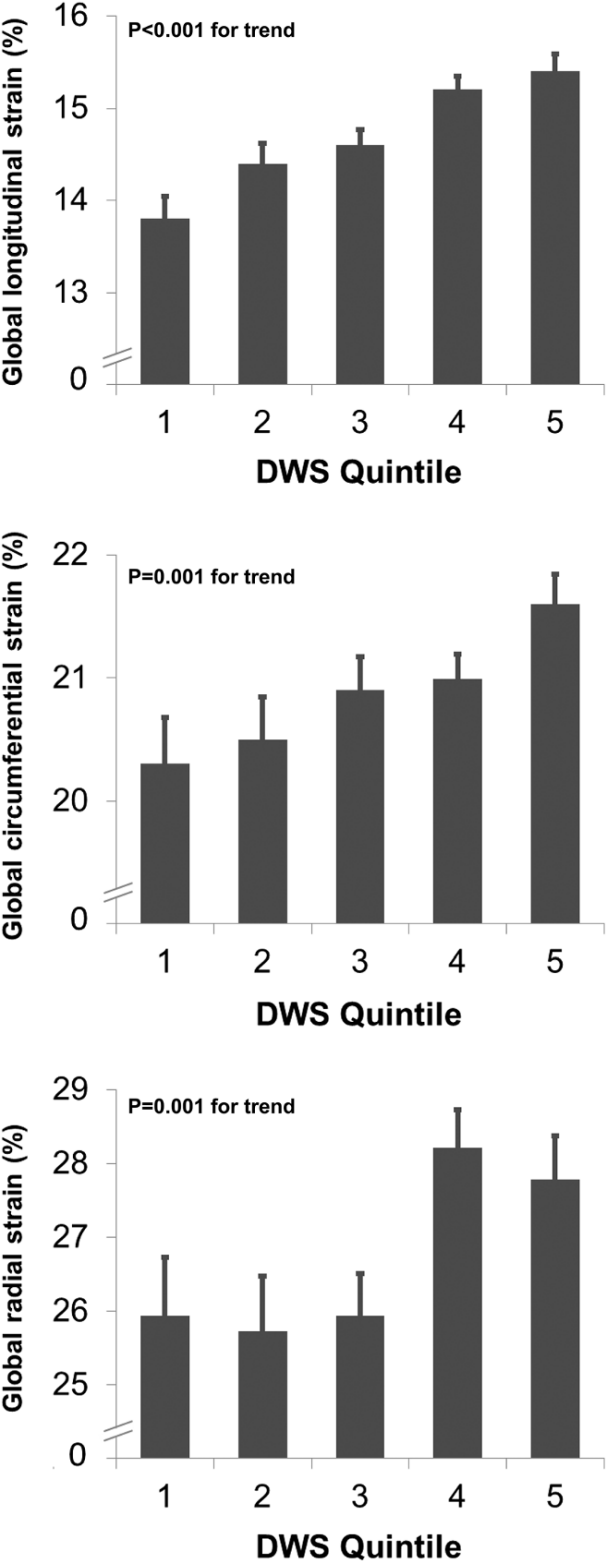
**Left ventricular systolic strain versus quintiles of diastolic wall strain in HyperGEN.** Bar graphs depict the relationship between diastolic wall strain and left ventricular global longitudinal strain, global circumferential strain, and global radial strain.

**Figure 2:**
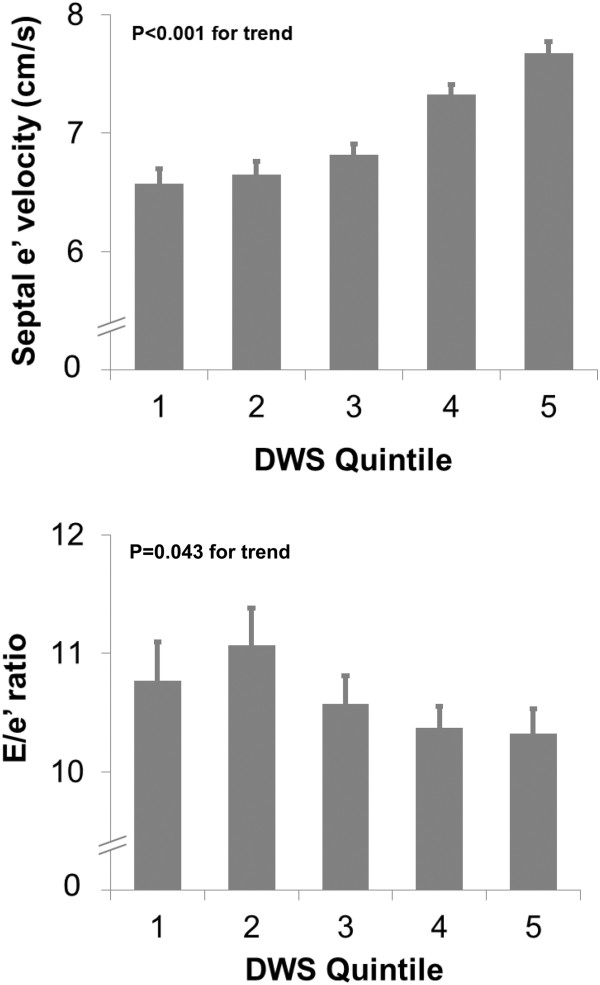
**Left ventricular diastolic indices versus quintiles of diastolic wall stiffness in HyperGEN.** Bar graphs depict the relationship between diastolic wall strain and left ventricular e’ velocity and E/e’ ratio.

Figures 1 and 2 show the relationship between DWS quintiles and LV strain parameters and diastolic indices, respectively. Table 3 demonstrates the correlation between DWS and both 2D and speckle-tracking echocardiographic systolic and diastolic indices. There was a modest but significant correlation between DWS and many systolic and diastolic indices. The strongest correlation was with midwall fractional shortening, a systolic index (R = 0.56, P < 0.001). DWS did not correlate with EF. Importantly, DWS did not correlate with measures of LV chamber compliance (EDV_20_ and early mitral inflow [E] deceleration time).

**Table 3:** **Correlation of diastolic wall strain with systolic and diastolic echocardiographic parameters in HyperGEN**

Echocardiographic parameter	Correlation coefficient	P-value
**Systolic indices**		
Global radial strain, %	0.07	0.002
Global circumferential strain, %	0.07	0.004
Global longitudinal strain, %	0.15	<0.001
Ejection fraction, %	0.03	0.19
Midwall shortening, %	0.56	<0.001
**Diastolic indices**		
e’ velocity, cm/s	0.23	<0.001
E/e’ ratio†	-0.07	0.03
Isovolumic relaxation time, ms	-0.19	<0.001
E/A ratio†, ‡	0.16	0.001
Early mitral inflow deceleration time, ms	-0.05	0.07
EDV_20_, mL*	-0.001	0.97

*EDV_*20*_ = left ventricular (LV) end-diastolic volume at an idealized LV end-diastolic pressure of 20 mmHg; †*E* = Early mitral inflow velocity; ‡*A* = Late (atrial) mitral inflow velocity.

### Independent association of DWS with cardiac mechanics in HyperGEN

Table 4 shows the association between DWS and cardiac mechanics using various linear mixed-effects models. DWS was associated with global longitudinal strain and e’ velocity on minimally-adjusted analysis (Model 1). After further adjustment for age, sex, LV mass-index, wall motion score index, and EF, DWS was associated with global radial strain, global circumferential strain, global longitudinal strain, and e’ velocity (Model 2). After additional adjustment for e’ velocity (Model 3) and E/e’ ratio (Model 4), DWS was still associated global longitudinal and circumferential strains.

**Table 4:** **Association of cardiac mechanics with diastolic wall strain in HyperGEN**

Dependent variable	Model 1*		Model 2 ^†^		Model 3 ^‡^		Model 4 ^§^	
	β-coefficient (95% CI)	P-value	β-coefficient (95% CI)	P-value	β-coefficient (95% CI)	P-value	β-coefficient (95% CI)	P-value
GRS,%	0.02 (-0.08, 0.12)	0.71	0.12 (0.003, 0.24)	0.049	0.12 (-0.01, 0.25)	0.06	0.16 (0.00, 0.32)	0.046
GCS,%	0.13 (-0.03, 0.06)	0.58	0.05 (0.003, 0.10)	0.037	0.06 (0.01, 0.11)	0.024	0.07 (0.02, 0.14)	0.020
GLS,%	0.10 (0.07, 0.12)	<0.001	0.07 (0.04, 0.10)	<0.001	0.05 (0.02, 0.08)	<0.001	0.07 (0.03, 0.11)	0.001
e’ velocity, cm/s	0.08 (0.07, 0.11)	<0.001	0.03 (0.01, 0.05)	<0.001	—	—	—	—
E/e’ ratio	-0.03 (-0.06, 0.01)	0.15	0.02 (-0.03, 0.06)	0.48	—	—	—	—

*Adjusted for speckle-tracking analyst, image quality, field center, and family relatedness.

†Adjusted for all covariates in Model 1 plus age, sex, left ventricular mass-index, wall motion score index, and ejection fraction.

‡Includes all covariates from Model 2 with additional adjustment for average tissue e’ velocity.

§Includes all covariates from Model 2 with additional adjustment for average E/e’ ratio.

*GRS* = Global radial strain; *GCS* = Global circumferential strain; *GLS* = Global longitudinal strain; *E* = Early mitral inflow velocity.

### Utility of DWS for the prediction of abnormal cardiac mechanics: validation study

**Figure 3:**
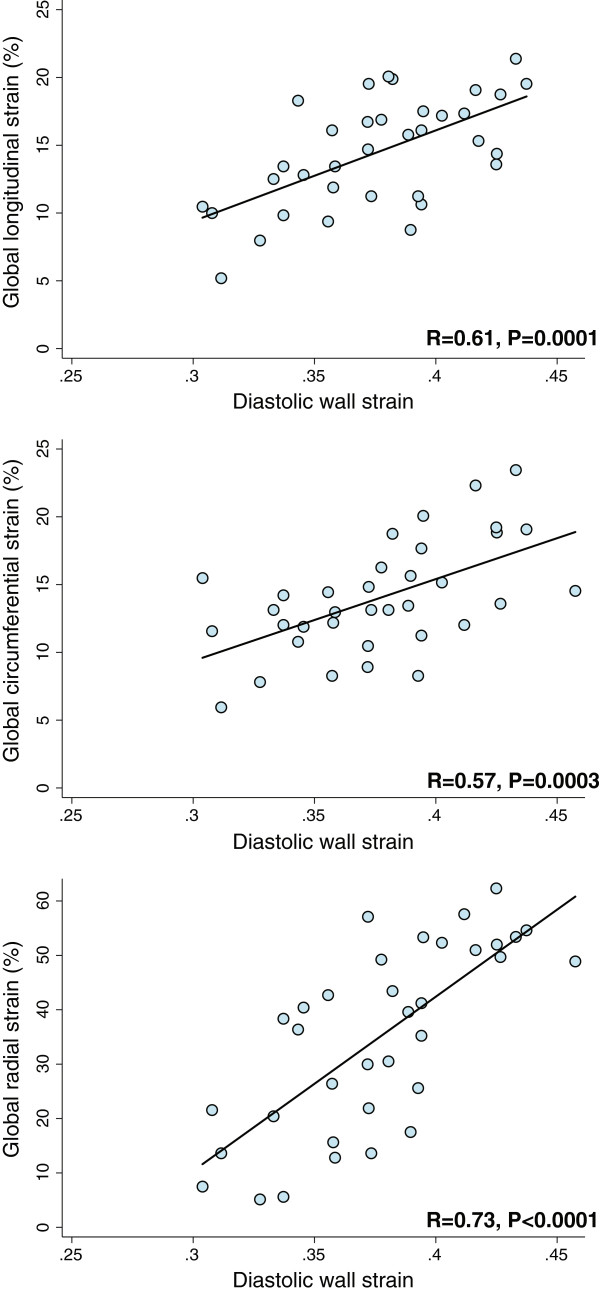
**Scatterplots demonstrating the correlation between diastolic wall strain and peak systolic strains measured prospectively on digital speckle-tracking analysis.** On prospective speckle-tracking analysis, DWS was most closely associated with global radial strain.

To determine the clinical utility of DWS as a predictor of abnormal myocardial strain, we conducted a prospective validation study using digital speckle-tracking echocardiography at high frame rates of 50–70 fps. Scatterplots depicting the relationship between DWS and peak systolic strains in this study are shown in Figure 3. We found that DWS (mean ± SD = 0.38 ± 0.04) correlated well with global longitudinal, circumferential, and radial strains, and was most highly correlated with global radial strain (Table 5). On ROC analysis, DWS demonstrated good discriminative ability (i.e., area under the ROC curve) for the detection of abnormal strain values (Table 5).

**Table 5:** **Prospective digital speckle-tracking analysis of diastolic wall strain as a marker of abnormal cardiac mechanics**

	Abnormal value	Correlation with DWS		ROC analysis
Strain parameter		R	P-value	C-statistic (95% CI)
Global longitudinal strain	< 12.5%	0.61	0.0001	0.78 (0.61-0.95)
Global circumferential strain	< 15%	0.57	0.0003	0.79 (0.61-0.97)
Global radial strain	< 35%	0.73	<0.0001	0.84 (0.71-0.97)

## Discussion

In one of the largest speckle-tracking studies to date, using echocardiograms from 1907 HyperGEN participants, we found that DWS correlated with both systolic and diastolic indices of LV performance and was independently associated with several indices of abnormal cardiac mechanics. These findings were significant after controlling for EF, LV mass index, and even diastolic indices of impaired relation (tissue e’ velocity) and filling pressures (E/e’ ratio). Our study is the first to comprehensively examine the association of DWS with systolic and diastolic echocardiographic parameters as well as the first to show the association of DWS with indices of systolic and diastolic cardiac mechanics. DWS, we conclude, is an overall marker of cardiac performance, including systolic and diastolic mechanics, but it is not a marker of LV diastolic chamber stiffness.

Few studies have examined the correlation of DWS with echocardiographic indices. The correlation between DWS and tissue e’ velocity has been confirmed [3]. Previous studies have disagreed on the correlation between DWS and E/e’ ratio [3, 4]. In our large study, however, we failed to find a correlation with E/e’. We also found no correlation between DWS and EDV_20_ or mitral inflow deceleration time, both established non-invasive markers of LV stiffness. We do, however, provide here the first evidence of significant correlations and associations between DWS and systolic echocardiographic indices (i.e., systolic strains) in humans. Interestingly, significant correlations between DWS and systolic parameters were not observed in an animal study, but this study consisted of only 25 rats and may have been underpowered [4].

Notably, Takeda et al., who first established the use of DWS to evaluate LV stiffness, did not find a correlation with tissue Doppler derived strain [4]. This may reflect the greater challenges of measuring strain with tissue Doppler technology versus speckle-tracking echocardiography, which is a direct marker of Lagrangian strain, less angle dependent, and more reproducible [5]. The association found in our study was indeed expected based on the mathematical formulations of both DWS and strain. However, in HyperGEN, DWS surprisingly did not have the strongest association with global radial strain, which is somewhat akin to DWS mathematically. However, it should be noted that DWS is measured from the posterior wall in the parasternal long axis and thus measures transverse strain, which differs from radial strain. In addition, the modest association between DWS and radial strain in HyperGEN likely reflects the greater difficulty in measuring global radial strain using speckle-tracking software. The radial motion of speckles acquired in the parasternal short axis view may fall out of line with the direction of the ultrasound beam, which causes poorer resolution of this motion and worse software tracking ability [20]. Based on our prospective digital speckle-tracking analysis of 35 patients, in which we found that DWS correlated best with radial strain, it is likely that technical issues are the primary reason for the only modest association between DWS and radial strain in HyperGEN.

Our study provides some insight into why DWS independently predicts adverse outcomes in patients with heart failure and preserved ejection fraction (HFpEF) [3]. Decreased DWS in HFpEF may be associated with poor outcomes through its association with abnormal systolic cardiac mechanics in addition to its association with abnormal diastolic cardiac mechanics. Other studies have also indicated that systolic abnormalities abound in HFpEF [21, 22], including reduced longitudinal, circumferential, and radial strain [23, 24]. Though not specifically studied in HFpEF patients, abnormalities in strain independently predict mortality [25], which raises the possibility that DWS predicts mortality in HFpEF perhaps as a marker of abnormal cardiac mechanics.

We therefore caution the clinical use of DWS as a pure marker of LV diastolic stiffness, since DWS is independently associated with abnormal systolic cardiac mechanics and correlates with both systolic and diastolic echocardiographic indices. The failure to DWS to correlate with other non-invasive measurements of LV stiffness is particularly remarkable. Why Takeda et al. were able to find a correlation between DWS and myocardial stiffness, while the present study did not, may be multifactorial. First, DWS is an abbreviated term from the original equation that sought to quantify LV stiffness, the epicardial motion index [(DWS)/(epicardial movement during diastole)] [4]. Though the epicardial motion index is a more exact marker of LV diastolic stiffness, this formula requires direct measurement of epicardial movement, which is difficult to achieve with 2D echocardiography. The epicardial motion index may better reflect LV diastolic stiffness compared to DWS, but its difficult implementation in routine clinical practice would reduce its clinical utility. Second, despite a predominantly hypertensive population, only 17% had echocardiographic evidence of LV hypertrophy, thereby reducing our ability to find a correlation between DWS and LV diastolic stiffness. Finally, the difference in findings may relate to technique of measurement of LV stiffness (invasive, catheter-based technique versus an non-invasive echocardiographic technique). Further research should be performed to determine more precisely the clinical utility of DWS.

Our results should be interpreted in the context of a few limitations. First, DWS is measured in the parasternal long axis view, whereas cardiac mechanics were measured in our study in the parasternal short axis and apical four chamber views. Thus, we did not assess myocardial mechanics precisely at the posterior wall in the parasternal long axis view where DWS is derived. Second, because HyperGEN echocardiographic data were collected at a time prior to tissue Doppler imaging, tissue velocities were recorded here using speckle-tracking analysis. However, we demonstrated that correlation between the two approaches is high and were able to convert our measurements into tissue Doppler values. Third, speckle-tracking was performed retrospectively on echocardiograms that were acquired without specific attention to optimizing endocardial border definition, a necessity for speckle-tracking software [5]. This may have accounted for the modest correlation coefficients for the relationship between DWS and systolic and diastolic parameters. However, the majority of images acquired were of at least adequate quality. In addition, image quality was entered into all regression analyses, and we purposely performed a prospective validation study to verify our findings in HyperGEN, and these data show that DWS is indeed clinically useful as an estimator of abnormal cardiac mechanics. Fourth, we are unable to calculate the myocardial performance index due to missing relevant data in the dataset, which should theoretically correlate with DWS if it is indeed an overall marker of myocardial function. Finally, our validation study was too small to reliably calculate clinically valid sensitivities and specificities (and optimal cut-off values) for DWS as a diagnostic test for abnormal systolic strain values. Further study will be necessary to determine whether DWS can be applied clinically.

## Conclusion

DWS correlates with both systolic and diastolic echocardiographic parameters. In addition, DWS is independently associated with multiple measures of systolic cardiac mechanics. DWS appears to reflect overall myocardial performance, and caution should be exercised clinically implementing this index to assess LV diastolic stiffness. Nevertheless, given its ease of use and widespread applicability, calculation of DWS could be a powerful new technique for the detection of subclinical cardiac disease.

## Abbreviations

2D: 2-dimensional
DWS: Diastolic wall strain
EDPVR: End-diastolic pressure-volume relationship
HFpEF: Heart failure with preserved ejection fraction
HyperGEN: Hypertension Genetic Epidemiology Network
LV: Left ventricle
PWTd: Posterior wall thickness during diastole
PWTs: Posterior wall thickness during systole.
